# Supplementary material for: Bacterial Culture Underestimates Lung Pathogen Detection and Infection Status in Cystic Fibrosis
Source: Microbiol Spectr. 2022 Aug 16;10(5):e00419-22. doi: 10.1128/spectrum.00419-22 (PMC9602735; doi:10.1128/spectrum.00419-22)
Supplement: Supplemental file 1 — Tables S1 to S3 and Fig. S1 and S2. Download spectrum.00419-22-s0001.pdf, PDF file, 1.4 MB [file spectrum.00419-22-s0001.pdf]

Supplementary materials

Bacterial culture underestimates lung pathogen detection and infection status in cystic fibrosis

Helen Gavillet[1], Lauren Hatfield[1], Damian Rivett[2], Andrew Jones[3,4], Anirban Maitra[5], Alexander Horsley[3,4,7], Christopher van der Gast[1,6,7 *]

[1] Department of Life Sciences, Manchester Metropolitan University, Manchester, UK

[2] Department of Natural Sciences, Manchester Metropolitan University, Manchester, UK

[3] Manchester Adult Cystic Fibrosis Centre, Manchester University NHS Foundation Trust, Manchester, UK

[4] Division of Infection, Immunity and Respiratory Medicine, University of Manchester, Manchester, UK

[5] Royal Manchester Children's Hospital, Manchester University NHS Foundation Trust, Manchester, Manchester, UK

[6] Department of Respiratory Medicine, Northern Care Alliance NHS Foundation Trust, Salford, UK

[7] Denotes senior authors of this work.

***Correspondence to:** Professor Chris van der Gast, Department of Life Sciences, Faculty of Science and Engineering, John Dalton Building, Chester Street, Manchester, M1 5GD, UK; C.vanderGast@mmu.ac.uk

Literature Review

PubMed was searched for studies on cystic fibrosis (CF) published from database inception to January 30th, 2022, with the search terms "cystic fibrosis" AND "pathogen" AND "culture" AND "QPCR", with no language restrictions (Table S1). Studies that did not use QPCR to target specific pathogens, that only used 16S rRNA gene targeted QPCR, only used next generation sequencing (NGS) approaches, or other detection methods such as dot blots were not considered in this literature review. Of the 21 papers that met the above criteria (Table S1), eight were cross-sectional only studies (1, 2, 5, 11, 12, 13, 19, 20). Of the remaining 13 papers that did incorporate some form of longitudinal sampling into their studies, ten studies pooled all samples for grouped analysis, irrespective of patients and time points that samples were taken from (3, 4, 6, 7, 9, 10, 16, 17, 18, 21).

The three remaining studies did explicitly track individual patients over time, but with caveats (8, 14, 15). One study (14), tracked individual patients but with a focus on the effectiveness of tracking *Pseudomonas aeruginosa* infection before it became apparent in routine clinical culture, and analysed data through pooled samples for a grouped average time to detection. A second study (15), focused on the impact of Ivacaftor on *P. aeruginosa* (PSA) along with the general microbiome via NGS. However, the analyses combined all patients as a group, discussing *P. aeruginosa* based results as an average of the group. The final paper (8), focused on similar issues to our study, i.e. whether QPCR would be more sensitive than conventional culture in the detection of PSA in individual patients through time. Comparison of targeted QPCR and culture was based chiefly on cough swab samples taken from only paediatric patients (n = 33) over eight months from 2007; mean number of samples per patient (± SD) = 4·2 ± 1·4, minimum = 1 and maximum = 7. The study found an increased detection of *P. aeruginosa* when using QPCR. Although pathogen detection was plotted for individual patients, data was analysed only as a group. Finally, none of the studies explicitly investigated the impact that more sensitive detection methods would have on infection status or clinical outcomes.

Table S1 Literature review comparing current study with results from PubMed search.

	No. patients	Paediatric	Adult	No. samples (sample type)	No. centres	Target pathogen [a]	QPCR primer target(s)[b]	Comparison to matched culture[c]	Study type	Duration (months)	Were individuals tracked?	Analysis (Grouped or Individual)[d]	Key takeaway points	Explicit clinical impact	Citation
	40	20	20	328 (234 sputum, 94 swabs)	2	PSA, SA	*PSA: oprL, SA: nuc*	Yes	Longitudinal	42 months	Yes	Individual	Comparison of culture data to QPCR, impact on clinical status for individual patients	Yes	This Study
**1**	Not clearly specified	n/a	n/a	200 (175 from sputum, 21 from swabs and 4 from BAL)	1	PSA	*oprL/ gyrB/ ETA*	Yes	Cross-sectional	No	n/a	Grouped	Combinations of primer targets will improve PSA detection	No	Qin X, 2003 (1)
**2**	8 patients	n/a	n/a	8 (sputum)	1	PSA	*oprL*	Yes	Cross-sectional	No	n/a	Grouped	Comparison of pre-treatments for samples to improve detection rates	No	Deschaught 2009 (2)
**3**	397	n/a	n/a	Unknown mix of sputum, nasopharyngeal or throat swab	7	PSA	*oprL*	Yes	Longitudinal	ca. 16 months	No	Grouped	Culture and QPCR work just as well as each other, the predictive value of QPCR may be quite limited	No	Deschaught 2010 (3)
**4**	33	33	0	103 (sputum)	1	PSA	*oprL*	Yes	Longitudinal	ca. 30 months	No	Grouped	QPCR can detect PSA before culture	No	Billard-Pomares 2010 (4)
**5**	Not clearly specified	n/a	n/a	n/a	Unknown	*Streptococcus spp.*	*cpn60*	No	Cross-sectional	No	n/a	Grouped	QPCR development	No	Olson 2010 (5)
**6**	16	n/a	n/a	159 (85 Sputum, 47 swabs and 27 saliva)	1	Various CF pathogens	*PSA: PSD7F, SA:STPYF, HI: unknown,*	Yes	Longitudinal	ca. 12 months	No	Grouped	Comparison of various pathogen primer targets	No	Zemanick 2010 (6)
**7**	183	183	0	2099 (851 sputum and 1248 throat swabs)	1	PSA	*gyrB/ algD*	Yes	Longitudinal	ca. 29 months	No	Grouped	QPCR can detect PSA before culture	No	Logan 2010 (7)
**8**	186	186	0	542 (42 sputum and 500 swabs	1	PSA	*Pa23*	Yes	Longitudinal	ca. 19 months	Yes	Grouped	QPCR is more sensitive than culture in PSA detection	Stated clinical impact needs assessing but doesn't go beyond that	McCulloch 2011 (8)
**9**	230	74	156	459 (293 sputum, 162 swab and 4 BAL)	2	PSA	*gyrB*	Yes	Longitudinal	ca. 30 months	1 patient tracked.	Grouped	Analysis of PSA levels during exacerbation & stability, with optimised method. No difference found in disease states	States introduction of QPCR may have implications on better segregation strategies and could help patients be free of PSA for longer.	Fothergill 2013 (9)
**10**	34	n/a	n/a	46 (sputum)	3	PSA	*oprL/ gyrB/ ecfX*	Yes	Longitudinal	ca. 38 months	No	Grouped	Prevention of false negatives using a 2-step confirmatory QPCR	No	Le Gall 2013 (10)

**11**	9	0	9	13 samples	1	PSA	*oprL* and *ClpX*	No	Cross-sectional	No	n/a	Grouped	Validation of 2 PSA reference genes in CF sputum	No	Costaglioli 2014 (11)
**12**	65	65	0	87 (45 sputum and 42 swabs)	1	SA	*femA*	Yes	Cross-sectional	No	n/a	Grouped	Method comparison for sample processing.	No	Johnson 2016 (12)
**13**	Not clearly specified	n/a	n/a	15 (11 sputum and 4 BAL)	Unknown	NTM	*atpE*	Yes	Cross-sectional	No	n/a	Grouped	Comparison of sample processing methods for better NTM detection.	No	Caverly 2016 (13)
**14**	96	96	0	707 (sputum)	1	PSA	*oprL/ gyrB/ ecfX*	Yes	Longitudinal	ca. 42 months	Yes	Grouped	QPCR can detect PSA before culture	No	Héry-Arnaud 2017 (14)
**15**	12	0	12	Not clearly specified	1	PSA, *Streptococcus, Prevotella*	*PSA: gyrB, SA: nuc*	Yes	Longitudinal	ca. 34 months	Yes	Grouped	Ivacaftor briefly impacts PSA levels, but PSA somewhat recovers over time.	No	Hisert 2017 (15)
**16**	64	n/a	n/a	379 (141 throat swabs and 238 sputum)	1	PSA	*oprL/ ecfX/ gyrB*	Yes	Longitudinal	Not clearly specified	No	Grouped	PSA abundance in swabs/sputum compared to culture, impact of mucoidity. Swabs may beneficial for early detection.	No	Boutin 2018 (16)
**17**	47	47	0	312 (sputum)	1	PSA	*oprL*	Yes	Longitudinal	ca. 17 months	No	Grouped	Time taken for PSA positive culture to be determined vs. QPCR	No	Blanchard 2018 (17)
**18**	Not clearly specified	n/a	n/a	88 (sputum)	1	PSA	*ecfX*	Yes	Longitudinal	ca. 20 months	No	Grouped	Comparison of sample storage, DNAse treatments and QPCR targets.	No	Mangiaterra 2018 (18)
**19**	9	0	9	16 (sputum)	1	SM	Custom primers for *Strenotrophomonas* strains	Yes	Cross-sectional	No	n/a	Grouped	QPCR development	Identification of SM in clinic will allow for earlier detection and by extension may reduce prevalence of other emblematic pathogens through correct treatment.	Fraser 2019 (19)
**20**	78	78	0	78 (BAL)	7	PSA, HI, SA, SM	Not clearly specified	Yes	Cross-sectional	No	n/a	Grouped	Correlation of bacterial density and presence of pathogens to inflammation, mucus plugging etc.	No	Taylor 2020 (20)
**21**	14	n/a	n/a	28 (sputum)	1	PSA	*oprL*	No	Longitudinal	ca. 12 months	Yes	Grouped	Does the prevalence of certain pathogens decrease with Ivacaftor treatment.	No	Einarsson 2021 (21)

[a] PSA: *Pseudomonas aeruginosa,* SA: *Staphylococcus aureus,* NTM: Non-tuberculosis mycobacteria, SM: *Streptococcus maltophilia, HI* : *Haemophilus influenzae*

[b] Papers using 16S rRNA, NGS or other methods such as dot blots were not considered in this literature review

[c] Must have matched culture records for each sample

[e] Explicitly followed individually through time, or grouped (all samples irrespective of longitudinal/cross-sectional origin, or combined for analysis)

Table S2 Individual patient sampling across the paediatric and adult cohorts. Given for each patient are the number of samples and date of each sample taken. Cells shaded in grey were taken during an acute pulmonary exacerbation. At entry to the study, all adults except for 3 patients were on long term azithromycin. Patients 133 and 135 were on no long-term antibiotic treatment, and patient 110 was on long term colistin. All but 6 paediatric patients were also on long term azithromycin, as patients 201, 206, 212, 216, 228 and 241 were not on any long-term antibiotic treatment.

Paediatric																				
201	09/12/14	31/03/15	21/07/15	27/10/15	16/02/16	21/06/16	30/08/16	29/08/17												
203	06/01/15	25/03/15	23/06/15	29/09/15	24/05/16	22/11/16	27/06/17	19/09/17	28/10/17											
206	16/01/15	03/07/15	29/01/16	06/09/16	09/12/16	02/06/17														
207	16/01/15	07/08/15	19/02/16	06/05/16	05/08/16	20/01/17	21/04/17													
212	26/05/15	25/08/15	08/09/15	09/02/16	18/10/16	01/08/17														
213	17/01/15	26/05/15	12/10/15	15/03/16	30/08/16	25/10/16	26/01/18													
216	07/08/15	06/11/15	15/01/16	06/05/16	11/11/16	13/01/17	07/07/17	29/09/17												
217	06/03/15	05/06/15	02/10/15	18/03/16	17/06/16	16/09/16	03/03/17	09/06/17												
218	10/03/15	04/08/15	26/01/16	06/09/16	09/12/16	02/06/17														
219	10/03/15	21/07/15	08/12/15	01/03/16	21/06/16	23/12/16														
223	29/09/15	15/03/16	15/11/16	23/05/17	17/10/17	23/01/18														
225	01/05/15	30/10/15	26/02/16	28/10/16	24/01/17	05/01/18														
228	12/05/15	04/08/15	24/11/15	12/04/16	27/09/16	04/04/17	04/07/17	10/10/17												
233	22/06/15	26/01/16	05/04/16	12/07/16	04/10/16	08/11/16														
235	10/07/15	24/07/15	06/08/15	09/10/15	08/01/16	11/03/16	09/12/16													
240	24/07/15	12/02/16	22/04/16	08/07/16	06/01/17	07/04/17	07/07/17	16/02/18												
241	24/07/15	23/10/15	22/01/16	11/03/16	08/09/17	01/12/17														
242	28/07/15	06/10/15	12/01/16	17/05/16	09/08/16	15/11/16	04/07/17	10/10/17												
245	04/08/15	30/08/15	03/11/15	26/01/16	09/08/16	08/11/16	10/10/17	23/01/18												
246	08/09/15	29/03/16	05/07/16	27/09/16	28/03/17	20/06/17	19/09/17	12/12/17												
Adult																				
101	21/11/14	15/12/14	26/01/15	23/02/15	18/05/15	29/06/15	24/08/15	19/10/15	14/12/15	25/01/16	04/04/16	16/05/16	27/06/16	15/08/16	03/10/16	07/11/16	16/01/17	24/04/17	02/10/17	29/01/18
103	24/11/14	23/02/15	20/07/15	14/09/15	14/12/15	14/11/16	23/01/17	10/04/17												
104	08/12/14	15/06/15	21/09/15	16/11/15	11/01/16	11/07/16	18/12/17													
106	01/12/14	12/01/15	30/03/15	22/06/15	03/08/15	07/09/15	02/11/15	23/05/16	03/10/16	03/04/17	21/08/17									
108	08/12/14	19/01/15	20/04/15	05/10/15	16/11/15	18/01/16	31/10/16	16/01/17	27/03/17	22/05/17	13/11/17	29/01/18								
110	23/02/15	27/04/15	01/06/15	14/12/15	18/04/16	23/01/17	03/08/17	30/10/17	05/02/18											
112	15/12/14	23/03/15	21/09/15	21/12/15	20/03/17	18/09/17	15/01/18													
113	15/12/14	19/01/15	16/02/15	16/03/15	18/05/15	01/02/16	16/05/16	06/06/16	25/07/16	08/08/16	19/09/16	04/12/17	15/01/18	05/02/18						
114	16/02/15	18/05/15	17/08/15	04/01/16	06/06/16	22/05/17	14/08/17	12/02/18												
115	02/03/15	27/04/15	17/08/15	28/09/15	02/11/15	04/01/16	12/09/16													
116	22/12/14	16/05/15	13/07/15	04/01/16	29/02/16	11/04/16	10/04/17													
118	05/01/15	16/03/15	30/11/15	11/01/16	09/05/16	20/06/16	28/11/16	20/02/17	15/05/17	19/06/17										
119	06/07/15	24/08/15	11/04/16	15/08/16	19/12/16	31/07/17	04/09/17	13/11/17	18/12/17	12/02/18										
120	26/01/15	20/04/15	20/07/15	26/10/15	25/01/16	07/03/16	09/05/16	20/06/16	12/09/16	03/04/17	04/12/17									
121	26/01/15	09/03/15	23/03/15	15/06/15	02/11/15	04/12/17														
132	27/07/15	25/01/16	25/04/16	09/05/16	13/06/16	10/04/17	24/07/17													
133	20/04/15	19/10/15	14/12/15	06/02/17	24/07/17	11/12/17														
135	18/05/15	13/07/15	07/09/15	19/10/15	13/03/17	13/11/17														
140	06/07/15	27/07/15	26/10/15	07/12/15	04/01/16	18/04/16	06/02/17	26/06/17	31/10/17	10/11/17	11/12/17	22/01/18								
147	05/12/16	09/01/17	06/03/17	24/04/17	15/05/17	19/06/17	10/07/17	14/08/17	18/09/17	04/12/17	29/01/18									

Table S3 Clinical characteristics of individual patients.

			CFTR Genotype			Pancreatic	Lung function [b]		CFTR modulator	Antibiotics received over course of study													
Patient	Age [a]	Sex	Genotype1	Genotype2	CF diabetes	insufficient	Mean %FEV_1	SD	therapy	Amoxicillin	Azithromycin	Ceftazidime	Ciprofloxacin	Co-amoxiclav	Colistin	Co-trimoxazole	Doxycycline	Flucloxacillin	Meropenem	Penicillin V	Promixin	Rifampicin	Tobramycin
101	22	Male	R334W	R75X	No	No	64.2	2.1	No		1			1		1	1	1	1				1
103	22	Male	F508del	3849+10kbC>T	No	No	79.7	7.9	No		1			1		1							
104	28	Male	3849+10kbC>T	3849+10kbC>T	No	No	77.9	7.5	No		1		1	1			1	1					
106	19	Male	F508del	F508del	No	Yes	57.2	8.0	No		1	1	1					1					1
108	35	Male	F508del	F508del	No	Yes	60.7	1.5	No		1		1										
110	28	Female	F508del	F508del	No	Yes	120.1	2.7	No		1	1	1	1	1								1
112	24	Male	F508del	F508del	No	No	93.2	4.5	No					1									
113	21	Female	F508del	Delexon2-3	No	Yes	58.1	1.4	No		1	1	1	1								1	1
114	21	Male	F508del	F508del	No	Yes	87.2	1.9	No		1			1									1
115	21	Female	F508del	F508del	No	Yes	47.9	2.2	No		1			1		1							
116	24	Male	R117H	S549N	No	No	99.6	2.1	No	1	1												
118	25	Female	F508del	G85E	No	Yes	73.8	4.2	No		1			1									
119	20	Female	R553X	2622+1G->A	Yes	Yes	47.9	2.9	No		1		1			1					1		
120	19	Male	F508del	F508del	No	Yes	78.6	3.3	No		1												
121	29	Male	F508del	R117H	No	No	92.9	3.7	No		1		1			1							
132	23	Female	F508del	G551D	No	Yes	105.7	2.3	Ivacaftor		1		1				1						
133	29	Male	F508del	2789+5G>A	No	Yes	80.7	6.6	No		1							1					
135	29	Female	N1303K	R117H	No	No	83.0	5.1	No					1									
140	17	Male	F508del	2622+1G>A	No	No	47.3	6.9	No		1			1		1	1						1
147	21	Female	F508del	M111R	No	Yes	71.4	2.7	No		1		1	1		1		1					
201	12	Female	F508del	G551D	No	Yes	84.4	2.7	Ivacaftor														
203	6	Female	F508del	F508del	No	Yes	100.2	10.7	No		1			1						1			
206	9	Female	G551D	1461ins4	No	Yes	91.1	6.6	Ivacaftor														
207	6	Female	1525-1G>A	1525-1G>A	No	Yes	95.7	6.0	No		1												
212	9	Female	F508del	F508del	No	Yes	83.9	6.3	No														
213	6	Male	F508del	F508del	No	Yes	82.3	15.7	No		1			1									
216	9	Female	S549N	S549N	No	Yes	117.8	5.2	No									1					
217	8	Male	F508del	G542X	No	Yes	100.4	2.7	No		1												
218	11	Male	F508del	F508del	No	Yes	89.6	6.6	No		1												
219	8	Male	F508del	R560T	No	Yes	98.7	1.4	No		1												
223	8	Male	F508del	S1235R	No	No	110.0	1.7	No		1												
225	12	Female	F508del	F508del	No	Yes	91.6	4.6	No		1												
228	6	Male	F508del	R553X	No	Yes	99.8	6.2	No														
233	15	Male	F508del	F508del	No	Yes	71.2	9.9	No		1												
235	15	Male	F508del	F508del	No	Yes	86.8	3.6	No		1												
240	11	Female	F508del	F508del	No	Yes	60.6	6.3	No		1												
241	8	Male	F508del	R117H	No	Yes	90.9	1.1	No														
242	6	Female	F508del	G551D	No	Yes	74.7	8.6	Ivacaftor		1												
245	6	Male	F508del	F508del	No	Yes	92.0	14.2	No		1	1											1
246	9	Female	3849+10kbC>T	3849+10kbC>T	No	No	91.0	9.1	No		1							1					

[a] Age in years at start of study. [b] Mean %FEV_1 and standard deviation of the mean (SD) over course of study.

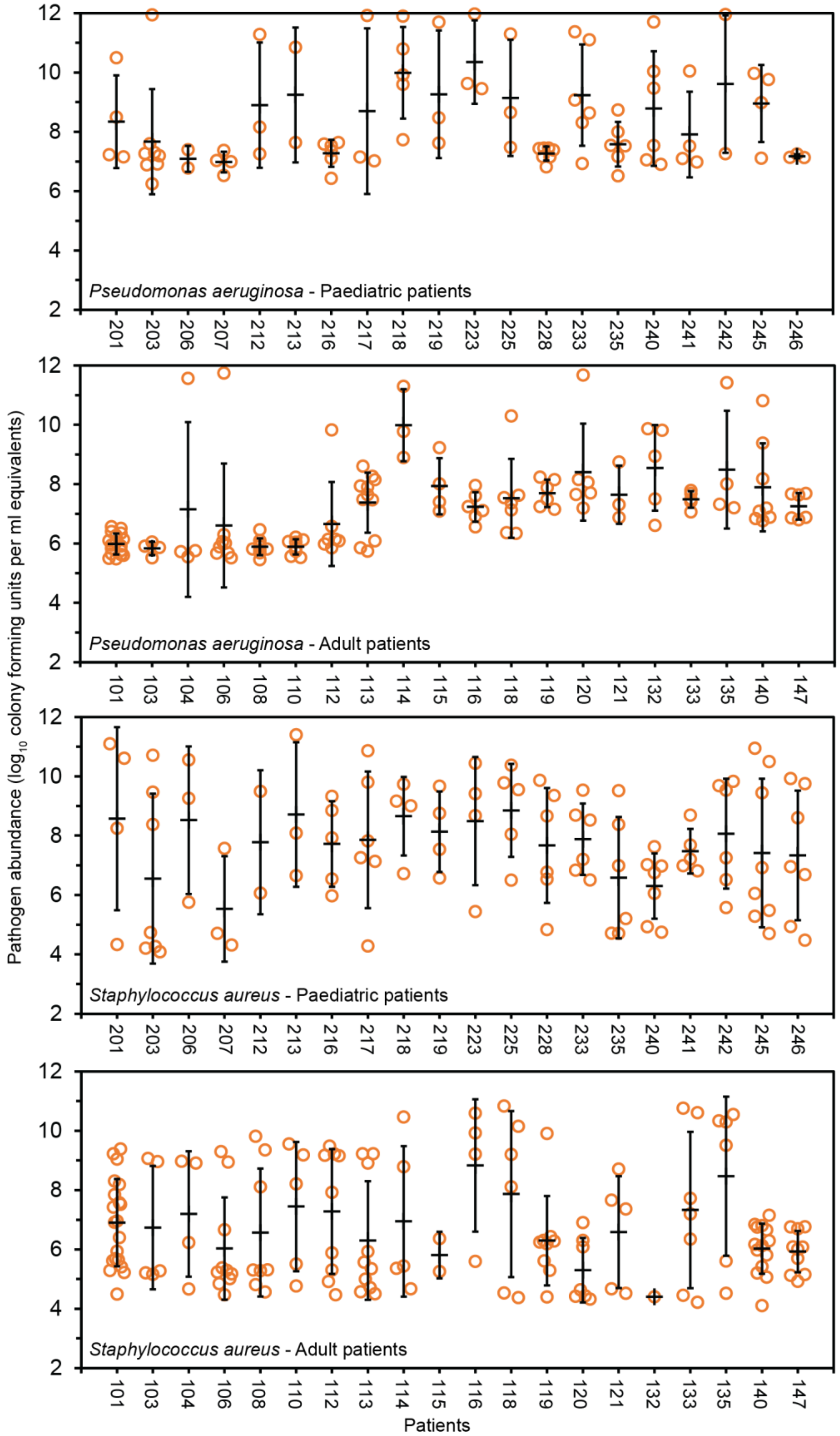

Figure S1 Pathogen abundance within paediatric and adult patients. Black lines indicate mean and standard deviation (SD) of the mean. Orange circles indicate individual samples for a given patient. For *P. aeruginosa*, the minimum and maximum abundances were 9.06×10^7 and 6.06×10^{10} colony forming units (CFU) ml^{-1} equivalents, respectively, in paediatric patients (mean ± SD, $1.91 \times 10^9 \pm 1.11 \times 10^1$), and ranged from 2.32×10^7 to 4.98×10^{10} CFU ml^{-1} equivalents in adult patients (mean ± SD, $4.75 \times 10^8 \pm 1.34 \times 10^1$). For *S. aureus*, abundance ranged from 1.29×10^6 to 3.70×10^{10} CFU ml^{-1} equivalents in paediatric patients (mean ± SD, $7.37 \times 10^8 \pm 2.54 \times 10^1$), and from 1.36×10^6 to 2.23×10^{10} CFU ml^{-1} equivalents in adult patients (mean ± SD, $1.85 \times 10^8 \pm 1.14 \times 10^1$). Lower detection limits derived from standard curves for *P. aeruginosa* and *S. aureus* were 2.2×10^2 and 5.2×10^3 CFU ml^{-1} equivalents, respectively.

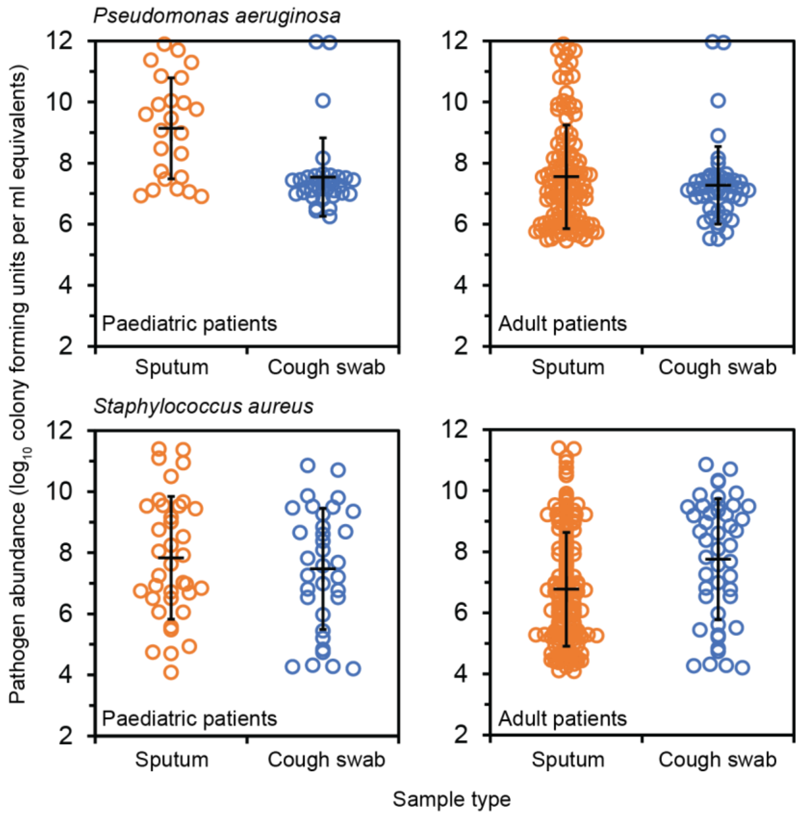

Figure S2 Comparison of pathogen abundance within paediatric and adult patients by underpinning respiratory sample type. Black lines indicate mean and standard deviation (SD) of the mean. Orange and blue circles indicate individual sputum and cough swab samples from an individual patient, respectively. For *P. aeruginosa* in paediatric patients, mean abundance from sputum and swab, respectively, was $4.08 \times 10^9 \pm 1.54 \times 10^2$ (n = 24) and $5{\cdot}97 \times 10^8 \pm 1.21 \times 10^1$ CFU ml^{-1} equivalents (n = 34); Kruskal-Wallis test, H =12.62, P <0.001. In adults, mean *P. aeruginosa* abundance from sputum and cough swabs was $6.05 \times 10^8 \pm 1{\cdot}97 \times 10^2$ (n = 123) and $4.13 \times 10^8 \pm 1{\cdot}10 \times 10^1$ CFU ml^{-1} equivalents (n = 48), respectively; Kruskal-Wallis test, H = 0.27, P = 0.601. For *S. aureus* in paediatric patients, sputum sample based mean abundance (± SD) was $8.71 \times 10^8 \pm 1.07 \times 10^3$ CFU ml^{-1} equivalents (n = 37) and from cough swabs was $5.42 \times 10^8 \pm 9.35 \times 10^2$ CFU ml^{-1} equivalents (n = 34); Kruskal-Wallis test, H = 0.39, P = 0.534. In adult patients, mean *S. aureus* abundance from sputum and cough swabs was $2.03 \times 10^8 \pm 5.06 \times 10^2$ (n = 157) and $7.90 \times 10^8 \pm 9.12 \times 10^2$ CFU ml^{-1} equivalents (n = 47), respectively; Kruskal-Wallis test, H = 1.62, P = 0.204. Lower detection limits derived from standard curves for *P. aeruginosa* and *S. aureus* were 2.2×10^2 and 5.2×10^3 CFU ml^{-1} equivalents, respectively.

References

1. Qin X, Emerson J, Stapp J, Stapp L, Abe P, Burns JL. 2003. Use of real-time PCR with multiple targets to identify *Pseudomonas aeruginosa* and other nonfermenting gram-negative bacilli from patients with cystic fibrosis. J Clin Microbiol 41:4312-7.
2. Deschaght P, De Baere T, Van Simaey L, Van Daele S, De Baets F, De Vos D, Pirnay JP, Vaneechoutte M. 2009. Comparison of the sensitivity of culture, PCR and quantitative real-time PCR for the detection of *Pseudomonas aeruginosa* in sputum of cystic fibrosis patients. BMC Microbiol 9:244.
3. Deschaght P, Schelstraete P, Lopes dos Santos Santiago G, Van Simaey L, Haerynck F, Van daele S, De Wachter E, Malfroot A, Lebecque P, Knoop C, Casimir G, Boboli H, Pierart F, Desager K, Vaneechoutte M, De Baets F. 2010. Comparison of culture and qPCR for the detection of *Pseudomonas aeruginosa* in not chronically infected cystic fibrosis patients. BMC Microbiology 10:245.
4. Billard-Pomares T, Herwegh S, Wizla-Derambure N, Turck D, Courcol R, Husson MO. 2011. Application of quantitative PCR to the diagnosis and monitoring of *Pseudomonas aeruginosa* colonization in 5-18-year-old cystic fibrosis patients. J Med Microbiol 60:157-161.
5. Olson AB, Sibley CD, Schmidt L, Wilcox MA, Surette MG, Corbett CR. 2010. Development of real-time PCR assays for detection of the *Streptococcus milleri* group from cystic fibrosis clinical specimens by targeting the cpn60 and 16S rRNA genes. J Clin Microbiol 48:1150-60.
6. Zemanick ET, Wagner BD, Sagel SD, Stevens MJ, Accurso FJ, Harris JK. 2010. Reliability of quantitative real-time PCR for bacterial detection in cystic fibrosis airway specimens. PLoS One 5:e15101.
7. Logan C, Habington A, Lennon G, Cronin F, O'Sullivan N. 2010. Evaluation of the efficacy of real-time polymerase chain reaction for the routine early detection of *Pseudomonas aeruginosa* in cystic fibrosis sputum and throat swab specimens. Diagn Microbiol Infect Dis 68:358-65.
8. McCulloch E, Lucas C, Ramage G, Williams C. 2011. Improved early diagnosis of *Pseudomonas aeruginosa* by real-time PCR to prevent chronic colonisation in a paediatric cystic fibrosis population. J Cyst Fibros 10:21-4.
9. Fothergill JL, Ledson MJ, Walshaw MJ, McNamara PS, Southern KW, Winstanley C. 2013. Comparison of real time diagnostic chemistries to detect *Pseudomonas aeruginosa* in respiratory samples from cystic fibrosis patients. J Cyst Fibros 12:675-681.
10. Le Gall F, Le Berre R, Rosec S, Hardy J, Gouriou S, Boisramé-Gastrin S, Vallet S, Rault G, Payan C, Héry-Arnaud G. 2013. Proposal of a quantitative PCR-based protocol for an optimal *Pseudomonas aeruginosa* detection in patients with cystic fibrosis. BMC Microbiol 13:143.
11. Costaglioli P, Barthe C, Fayon M, Christoflour N, Bui S, Derlich L, Domblides P, Crouzet M, Vilain S, Garbay B. 2014. Selection of *Pseudomonas aeruginosa* reference genes for RT-qPCR analysis from sputum of cystic fibrosis patients. Mol Cell Probes 28:10-2.
12. Johnson EJ, Zemanick ET, Accurso FJ, Wagner BD, Robertson CE, Harris JK. 2016. Molecular identification of *Staphylococcus aureus* in airway samples from children with cystic fibrosis. PLoS One 11:e0147643.
13. Caverly LJ, Carmody LA, Haig SJ, Kotlarz N, Kalikin LM, Raskin L, LiPuma JJ. 2016. Culture-independent identification of nontuberculous Mycobacteria in cystic fibrosis respiratory samples. PLoS One 11:e0153876.
14. Héry-Arnaud G, Nowak E, Caillon J, David V, Dirou A, Revert K, Munck MR, Frachon I, Haloun A, Horeau-Langlard D, Le Bihan J, Danner-Boucher I, Ramel S, Pelletier MP, Rosec S, Gouriou S, Poulhazan E, Payan C, Férec C, Rault G, Le Gal G, Le Berre R. 2017. Evaluation of quantitative PCR for early diagnosis of *Pseudomonas aeruginosa* infection in cystic fibrosis: a prospective cohort study. Clin Microbiol Infect 23:203-207.
15. Hisert KB, Heltshe SL, Pope C, Jorth P, Wu X, Edwards RM, Radey M, Accurso FJ, Wolter DJ, Cooke G, Adam RJ, Carter S, Grogan B, Launspach JL, Donnelly SC, Gallagher CG, Bruce JE, Stoltz DA, Welsh MJ, Hoffman LR, McKone EF, Singh PK. 2017. Restoring cystic fibrosis transmembrane conductance regulator function reduces airway bacteria and

inflammation in people with cystic fibrosis and chronic lung infections. Am J Respir Crit Care Med 195:1617-1628.
16. Boutin S, Weitnauer M, Hassel S, Graeber SY, Stahl M, Dittrich AS, Mall MA, Dalpke AH. 2018. One time quantitative PCR detection of *Pseudomonas aeruginosa* to discriminate intermittent from chronic infection in cystic fibrosis. J Cyst Fibros 17:348-355.
17. Blanchard AC, Rooney AM, Yau Y, Zhang Y, Stapleton PJ, Horton E, Klingel M, Stanojevic S, Ratjen F, Coburn B, Waters V. 2018. Early detection using qPCR of *Pseudomonas aeruginosa* infection in children with cystic fibrosis undergoing eradication treatment. J Cyst Fibros 17:723-728.
18. Mangiaterra G, Amiri M, Di Cesare A, Pasquaroli S, Manso E, Cirilli N, Citterio B, Vignaroli C, Biavasco F. 2018. Detection of viable but non-culturable *Pseudomonas aeruginosa* in cystic fibrosis by qPCR: a validation study. BMC Infect Dis 18:701.
19. Fraser TA, Bell MG, Harris PNA, Bell SC, Bergh H, Nguyen TK, Kidd TJ, Nimmo GR, Sarovich DS, Price EP. 2019. Quantitative real-time PCR assay for the rapid identification of the intrinsically multidrug-resistant bacterial pathogen *Stenotrophomonas maltophilia*. Microb Genom 5.
20. Taylor SL, Leong LEX, Ivey KL, Wesselingh S, Grimwood K, Wainwright CE, Rogers GB. 2020. Total bacterial load, inflammation, and structural lung disease in paediatric cystic fibrosis. J Cyst Fibros 19:923-930.
21. Einarsson GG, Ronan NJ, Mooney D, McGettigan C, Mullane D, NiChroinin M, Shanahan F, Murphy DM, McCarthy M, McCarthy Y, Eustace JA, Gilpin DF, Elborn JS, Plant BJ, Tunney MM. 2021. Extended-culture and culture-independent molecular analysis of the airway microbiota in cystic fibrosis following CFTR modulation with ivacaftor. J Cyst Fibros 20:747-753.
